# Breakdown of the effective medium theory: a perspective from Goos–Hänchen shift

**DOI:** 10.1515/nanoph-2025-0314

**Published:** 2025-09-05

**Authors:** Wenqian Gong, Yiyu Shi, Zhenxing Liu, Chi Zhang, Zhiwei Cui, Yu Chen, Xinxing Zhou

**Affiliations:** Key Laboratory of Low-Dimensional Quantum Structures and Quantum Control of Ministry of Education, School of Physics and Electronics, 12568 Hunan Normal University, Changsha 410081, China; School of Physics, Xidian University, Xi’an, 710071, China; International Collaborative Laboratory of 2D Materials for Optoelectronics Science and Technology, Institute of Microscale Optoelectronics, Shenzhen University, Shenzhen 518060, China; Key Laboratory of Physics and Devices in Post-Moore Era, College of Hunan Province, Changsha 410081, China; Hunan Research Center of the Basic Discipline for Quantum Effects and Quantum Technologies, Hunan Normal University, Changsha 410081, China

**Keywords:** effective medium theory, Goos–Hänchen shift, breakdown, nanoscale sensing

## Abstract

The effective medium theory (EMT) provides a simplified framework to calculate the electromagnetic responses and is generally considered exact in the all-dielectric system with deep-subwavelength constituents. In this work, we perform the Goos–Hänchen (GH) shift that invalidates the EMT on the multilayered dielectric structures under the common conditions. This breakdown of the EMT arises from the high sensitivity of the GH shift on the phase and magnitude of Fresnel reflection coefficient. The degree of such breakdown shows strong dependence on the polarization angle of incidence and the layer and filling fraction of the structures. Notably, we find that the GH shift is potentially applicable to nano-meter scale thickness sensing, which cannot be displayed based on EMT in some cases. Our findings will provide useful guidance to reduce the calculation errors of the electromagnetic responses and promote the design of precise metrology devices.

## 1 Introduction

The calculation of the electromagnetic responses in the subwavelength-sized inhomogeneous structures is complex and difficult. To solve this problem, the effective medium theory (EMT) is proposed [1], [2]. Its conceptual process involves replacing the inhomogeneous structures with a homogeneous “effective medium” with uniform properties. This provides the fundamental approximation method to obtain the same response as the complex inhomogeneous structures to the identical excitation. In practice, its two predominant frameworks are the Maxwell–Garnett EMT [3] and Bruggeman EMT [4]. The former is applied in reliable predictions for electro-magnetic properties in a continuous host medium containing sparse inclusions at low volume fractions. In contrast, the latter enables precise modeling of the dielectric permittivity and conductivity of the high-concentration systems near the percolation threshold. To date, the EMT has been widely used in the description of the index of permittivity [5], [6], [7], permeability [8], [9], and surface conductivity [10], [11], [12] of complex materials, to name a few. Importantly, the effective medium can exhibit the extraordinary characteristics such as birefringence [13], Anderson localization [14], negative refractive index [15], [16], [17], and near-zero permittivity [18], [19], and leads to the significant findings like the metasurface [20], [21], [22], cloak [23], [24], and super-lens [25], [26], [27].

Despite its extensive applications, the EMT will be invalid in some cases. Typically, the EMT fails to describe the homogenization of the periodic metal-dielectric structures involving the large wave vectors, especially when the metal plasmons and hyperbolic high-*k* modes exist [28], [29], [30]. The underlying mechanism behind this phenomenon is ascribed to the inappropriate homogenization of the local structure properties that can strongly influence the overall structure properties. Therefore, the all-dielectric systems that do not support the large wave vectors are believed to be adaptable to the EMT for a long time. In recent years, it has been found that the EMT will also be invalid in the all-dielectric systems [31], [32], [33], [34], [35]. Sheinfux et al. first theoretically proposed the breakdown of EMT in a purely dielectric structure near the critical angle [31], which was later experimentally verified by Zhukovsky et al. [32]. Furthermore, the similar phenomena for the evanescent waves are successively predicted and observed in the one-dimensional disordered deep-subwavelength optical system due to the Anderson localization [33], [34]. Lately, a fundamental breakdown of the EMT by the photonic spin Hall effect in all-dielectric systems has been reported [35]. It stems from the spin–orbit interaction of light that is sensitive to the slight phase accumulations during the wave propagation. These works reveal that, not only the magnitude, but also the phase deviation of Fresnel reflection coefficients calculated by EMT may have great impacts on the final electromagnetic response. The Goos–Hänchen (GH) shift is the longitudinal beam shift in the incident plane [36], and attracted intensive attention due to its potential applications in precise sensing [37], [38], [39]. Besides, it is usually studied in the framework of EMT [40], [41], [42]. Now, an interesting question arises: as the typical electromagnetic response associated with the Fresnel reflection coefficient phase and magnitude, will the GH shift invalidate the EMT in the general all-dielectric system?

To address this question, we calculate the spatial and angular GH shifts on the all-dielectric periodic multilayered structures via the EMT and transfer matrix method (TMM), respectively, in this work. Numerical results show that the breakdown of EMT will emerge under the general conditions even without the critical angle. The physical mechanism is that the GH shift is very sensitive to the phase and magnitude of the Fresnel reflection coefficient. Besides, we examine the effects of the polarization angle of the incidence and the layer and filling fraction of the structures on the degree of EMT breakdown. Finally, we reveal the potential application of the GH shift on the detection of structure defects at the extreme nanoscale, which cannot be demonstrated in the EMT framework in some cases. These results can deepen the understanding of the EMT in various application realms and further the design of the relevant devices.

## 2 Theoretical model and numerical method

**Figure 1:**
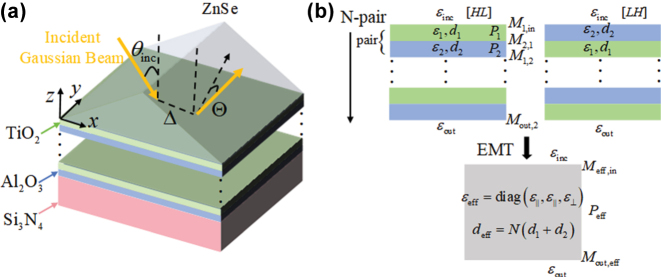
Illustrations of the GH shifts and the EMT. (a) Schematic of an all-dielectric periodic multilayered structure containing low-index (*L*) layers made of alumina (Al_2_O_3_), high-index (*H*) layers made of titania (TiO_2_), a superstrate made of zinc selenide (ZnSe), and a substrate made of silicon nitride (Si_3_N_4_), and the spatial Δ and angular Θ GH shifts of the Gaussian beam with the incident angle *θ*_*i*_. (b) Schematic of the rigorous transfer matrix method (TMM) with the different stacking orderings [*LH*] and [*HL*], and effective medium theory (EMT).

We will illustrate our idea by considering an all-dielectric periodic multilayered structure composed of *N* pairs of TiO_2_ and Al_2_O_3_ with permittivities *ɛ*_1_ and *ɛ*_2_, and thicknesses *d*_1_ and *d*_2_, respectively, and the superstrate ZnSe and substrate Si_3_N_4_ with permittivities *ɛ*_inc_ and *ɛ*_out_, as shown in Figure 1(a). The Fresnel reflection and refraction coefficients of such a structure can be rigorously calculated by the TMM as [43]

(1) $$\begin{array}{cc} \left[\begin{array}{c} t\\ 0 \end{array}\right] & =M_{\text{out,}2}P_{2}{\left(M_{2\text{,}1}P_{1}M_{1,2}P_{2}\right)}^{N-1}M_{2,1}P_{1}M_{1\text{,inc}}\left[\begin{array}{c} 1\\ r \end{array}\right]\\ & =Q^{\text{TMM}}\left[\begin{array}{c} 1\\ r \end{array}\right], \end{array}$$

with the converting *M* and propagating *P* matrix expressed as

(2) $$M_{i,j}=\frac{1}{2}\left[\begin{array}{cc} 1+\Delta_{i,j} & 1-\Delta_{i,j}\\ 1-\Delta_{i,j} & 1+\Delta_{i,j} \end{array}\right],\;P_{i}=\left[\begin{array}{cc} {\text{e}}^{\text{i}k_{iz}d_{i}} & 0\\ 0 & e^{-ik_{iz}d_{i}} \end{array}\right],$$

where $k_{iz}=k_{0}\sqrt{\varepsilon_{i}-\varepsilon_{\text{in}}\sin^{2}\theta_{i}}$ , Δ_*i*,*j*_ = *k*_*jz*_/*k*_*iz*_ and $\Delta_{i,j}=\varepsilon_{j}k_{iz}/\left(\varepsilon_{i}k_{jz}\right)$ for *s*- and *p-*polarized incidence, respectively, subscript *i* and *j* stand for the *i*-th and *j*-th layer, *k*_0_ is the wave number in vacuum, and *θ*_*i*_ is the incident angle. Finally, the *r* ^TMM^ and *t* ^TMM^ can be written as

(3) $$r^{\text{TMM}}=-\frac{Q_{21}^{\text{TMM}}}{Q_{22}^{\text{TMM}}},\;t^{\text{TMM}}=Q_{11}^{\text{TMM}}-\frac{Q_{12}^{\text{TMM}}Q_{21}^{\text{TMM}}}{Q_{22}^{\text{TMM}}}.$$

For the EMT illustrated in Figure 1(b), such structure can be described as an effective uniaxial slab with relative permittivity $\bar{\bar{\varepsilon }}=\mathrm{diag}\left(\varepsilon_{\|},\varepsilon_{\|},\varepsilon_{⊥}\right)$ with [1], [2]

(4) $$\varepsilon_{\|}=\frac{\varepsilon_{1}d_{1}+\varepsilon_{2}d_{2}}{d_{1}+d_{2}},\;\varepsilon_{⊥}=\frac{\varepsilon_{1}\varepsilon_{2}\left(d_{1}+d_{2}\right)}{\varepsilon_{1}d_{2}+\varepsilon_{2}d_{1}}.$$

The Fresnel reflection and refraction coefficients between the superstrate and slab are

(5) $$\left[\begin{array}{c} t\\ 0 \end{array}\right]=M_{\text{out,eff}}P_{\text{eff}}M_{\text{eff,inc}}\left[\begin{array}{c} 1\\ r \end{array}\right]=Q^{\text{EMT}}\left[\begin{array}{c} 1\\ r \end{array}\right],$$

where the matrices *M* and *P* have the similar forms as that in Eq. (2). Δ_out,eff_ = *k*_eff*z*_/*k*_out*z*_ and $\Delta_{\text{out},\text{eff}}=\varepsilon_{\|}k_{\text{out}z}/\left(\varepsilon_{\text{out}}k_{\text{eff}z}\right)$ , $k_{\text{eff}z}=k_{0}\sqrt{\varepsilon_{\|}-\varepsilon_{\text{inc}}\sin^{2}\theta_{\text{inc}}}$ and $k_{\text{eff}z}=k_{0}\sqrt{\varepsilon_{⊥}-\varepsilon_{\text{inc}}\sin^{2}\theta_{\text{inc}}}$ , and Δ_eff,inc_ = *k*_inc*z*_/*k*_eff*z*_ and $\Delta_{\text{eff,inc}}=\varepsilon_{\text{inc}}k_{\text{eff}z}/\left(\varepsilon_{\|}k_{\text{inc}z}\right)$ , for *s*- and *p-*polarized incidence, respectively. Meanwhile, the effective thickness $d_{\text{eff}}=N\left(d_{1}+d_{2}\right)$ . Similarly, the *r* ^EMT^ and *t* ^EMT^ can be obtained as

(6) $$r^{\text{EMT}}=-\frac{Q_{21}^{\text{EMT}}}{Q_{22}^{\text{EMT}}},\;t^{\text{EMT}}=Q_{11}^{\text{EMT}}-\frac{Q_{12}^{\text{EMT}}Q_{21}^{\text{EMT}}}{Q_{22}^{\text{EMT}}}.$$

Next, we proceed to derive the GH shift for reflection, i.e., the longitudinal centroid shift of the reflected beam. The vector angular spectrum of the incident Gaussian beam is [44]

(7) $${\tilde{E}}_{i}=\left[\cos\beta {\hat{x}}_{i}+\sin\beta {\hat{y}}_{i}-\frac{1}{k_{0}}\left(\cos\beta k_{ix}+\sin\beta k_{iy}\right){\hat{z}}_{i}\right]{\tilde{u}}_{i},$$

with

(8) $${\tilde{u}}_{i}\left(k_{ix},k_{iy}\right)=\frac{w_{0}^{2}}{4\pi }e^{-\left(k_{ix}^{2}+k_{iy}^{2}\right)w_{0}^{2}/4},$$

where *β* is the polarization angle, *w*_0_ is the beam waist, and *k*_*ix*_ and *k*_*iy*_ are wave vector components along the *x*_*i*_ and *y*_*i*_ axes, respectively, in local coordinates $\left(x_{i},y_{i},z_{i}\right)$ for incidence. Based on the three-dimensional beam propagation model, the vector angular spectrum of the reflected Gaussian beam is expressed as

(9) $$\begin{array}{ll} {\tilde{E}}_{r} & =\left[\cos\beta \left(r_{p}-\frac{\partial r_{p}}{\partial \theta_{\text{inc}}}\frac{k_{rx}}{k_{0}}\right)+\sin\beta \left(r_{p}+r_{s}\right)\frac{k_{ry}}{k_{0}}\cot\theta_{i}\right]\\ & \;\times {\tilde{u}}_{r}{\hat{x}}_{r}\;+\left[\sin\beta \left(r_{s}-\frac{\partial r_{s}}{\partial \theta_{\text{inc}}}\frac{k_{rx}}{k_{0}}\right)\right.\\ & \left.\;-\cos\beta \left(r_{p}+r_{s}\right)\frac{k_{ry}}{k_{0}}\cot\theta_{i}\right]{\tilde{u}}_{r}{\hat{y}}_{r}\;\\ & \;-\frac{1}{k_{0}}\left(\cos\beta r^{p}k_{rx}+\sin\beta r^{s}k_{ry}\right){\tilde{u}}_{r}{\hat{z}}_{r}, \end{array}$$

in which *k*_*rx*_ and *k*_*ry*_ are wave vector components in local coordinates $\left(x_{i},y_{i},z_{i}\right)$ for reflection and satisfying the relationships *k*_*rx*_ = −*k*_*ix*_, *k*_*ry*_ = *k*_*iy*_, and ${\tilde{u}}_{r}\left(k_{rx},k_{ry}\right)={\tilde{u}}_{i}\left(-k_{ix},k_{iy}\right)$ .

The GH shift at any given plane *z* = const can be given by

(10) $$\left\langle x\right\rangle =\Delta +z\Theta =\frac{\left\langle {\tilde{E}}_{r}\right|i\partial_{k_{rx}}\left|{\tilde{E}}_{r}\right\rangle }{\left\langle {\tilde{E}}_{r}\left|\right.{\tilde{E}}_{r}\right\rangle }+\frac{z}{\sqrt{\varepsilon_{\text{inc}}}k_{0}}\frac{\left\langle {\tilde{E}}_{r}\right|k_{rx}\left|{\tilde{E}}_{r}\right\rangle }{\left\langle {\tilde{E}}_{r}\left|\right.{\tilde{E}}_{r}\right\rangle },$$

with $\partial_{k_{rx}}={\hat{x}}_{r}\partial /\partial k_{rx}$ and $k_{rx}={\hat{x}}_{r}k_{rx}$ . Finally, the spatial (Δ) and angular (Θ) GH shifts in reflection of the arbitrary linearly polarized incident Gaussian beam can be derived as

(11) $$\begin{array}{c} \Delta =\frac{1}{\sqrt{\varepsilon_{\text{inc}}}k_{0}}\frac{{\left|\cos\beta r_{p}\right|}^{2}\frac{\partial \phi_{p}}{\partial \theta_{\text{inc}}}+{\left|\sin\beta r_{s}\right|}^{2}\frac{\partial \phi_{s}}{\partial \theta_{\text{inc}}}}{{\left|\cos\beta r_{p}\right|}^{2}+{\left|\sin\beta r_{s}\right|}^{2}},\\ \Theta =-\frac{2}{\varepsilon_{\text{inc}}k_{0}^{2}w_{0}^{2}}\frac{{\left|\cos\beta \right|}^{2}\frac{\partial {\left|r_{p}\right|}^{2}}{\partial \theta_{\text{inc}}}+{\left|\sin\beta \right|}^{2}\frac{\partial {\left|r_{s}\right|}^{2}}{\partial \theta_{\text{inc}}}}{{\left|\cos\beta r_{p}\right|}^{2}+{\left|\sin\beta r_{s}\right|}^{2}}, \end{array}$$

where *β* is the polarization angle (*β* = 0 and *β* = 90° denote the *p*- and *s*-polarization, respectively), and *ϕ*_*p*,*s*_ is the phase of *r*_*p*,*s*_. Notably, the expression of the spatial (Δ) GH shift derived by the stationary-phase analysis applies only when the reflection coefficient phase varies slowly.

## 3 Results and discussion

In the following numerical simulations, if no otherwise stated, we assume that the permittivities *ɛ*_1_ and *ɛ*_2_ of TiO_2_ and Al_2_O_3_ are 6.67 [45] and 3.12 [46], respectively, the thicknesses *d*_1_ and *d*_2_ of TiO_2_ and Al_2_O_3_ are 10 nm, permittivities *ɛ*_inc_ and *ɛ*_out_ of superstrate ZnSe and substrate Si_3_N_4_ are 6.65 [47] and 4.04 [48], respectively, the periodicity number of the structure *N*_pair_ = 10, the stacking orderings of the multilayers is [*HL*], the polarization angle *β* = 0, the wavelength of incidence in vacuum is 0.6328 μm, and the beam waist of incidence is 27 μm. Besides, we emphasize that the results calculated by TMM are exact.

**Figure 2:**
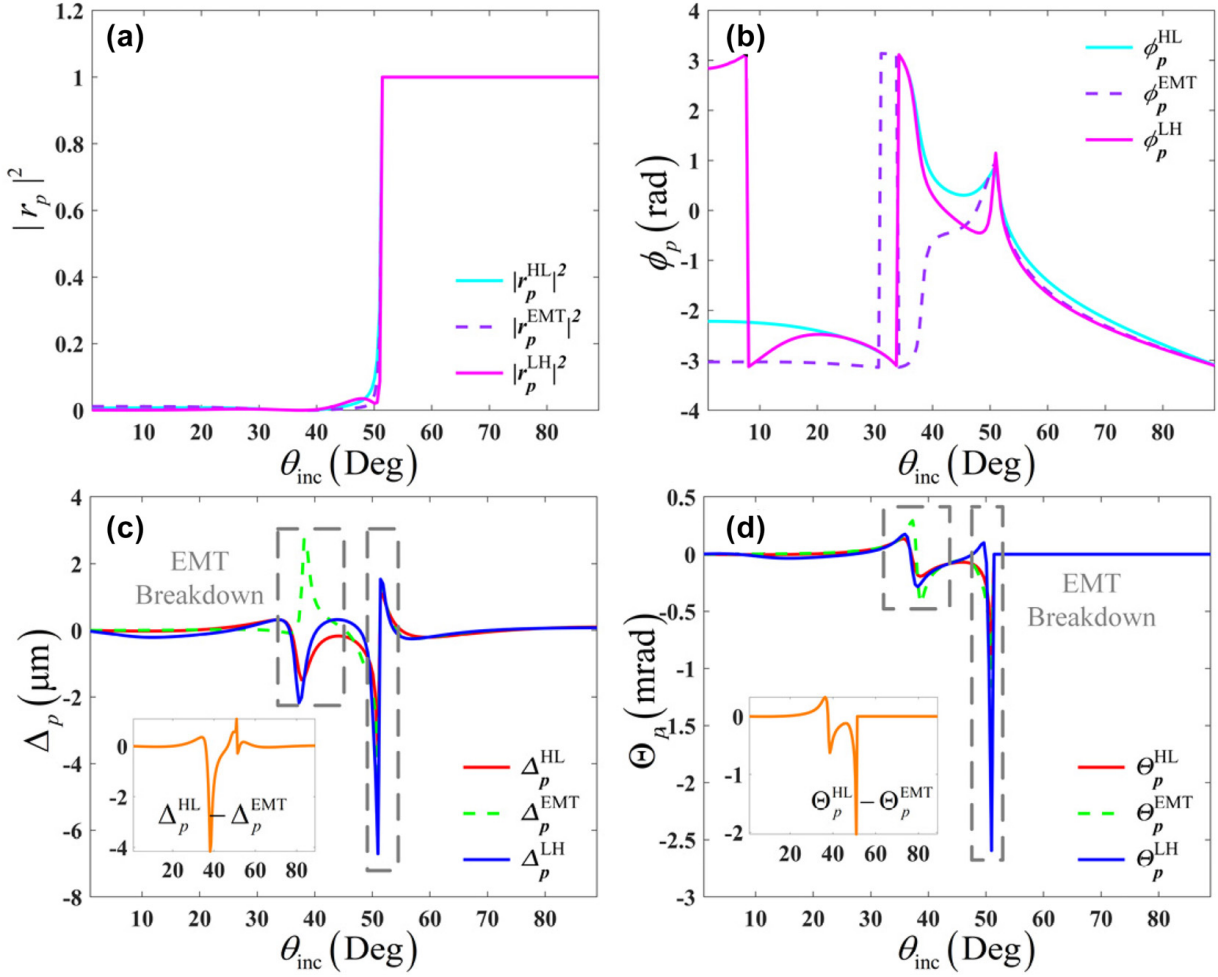
Breakdown of the EMT by the spatial (Δ) and angular (Θ) GH shifts on the all-dielectric periodic multilayered structures with different stacking orderings [*LH*] and [*HL*], in the case of *ɛ*_inc_ = 6.65. (a) Reflectivity |*r*_*p*_|^2^, (b) Fresnel reflection coefficient phase *ϕ*_*p*_, (c) spatial GH shifts (Δ), and (d) angular GH shifts (Θ).

First, we check the invalidity of the EMT in the structure shown in Figure 1(a), via the performance of spatial and angular GH shifts. Under this scenario, the critical angle 51.23° exists at the interface between the superstrate and effective medium. Figure 2 illustrates breakdown of the EMT by the spatial and angular GH shifts on the all-dielectric periodic multilayered structures with different stacking orderings [*LH*] and [*HL*], in case of *ɛ*_inc_ = 6.65. According to the EMT, such a multilayered structure of both [*LH*] and [*HL*] will be homogenized to the same effective medium, which means the $\Delta_{p}^{\text{EMT}}$ ( $\Theta_{p}^{\text{EMT}}$ ) should be equal to the $\Delta_{p}^{\text{HL}}$ ( $\Theta_{p}^{\text{HL}}$ ) and $\Delta_{p}^{\text{LH}}$ ( $\Theta_{p}^{\text{LH}}$ ). However, large discrepancies are found in these spatial and angular GH shifts. The discrepancies of angular GH shifts near the critical angle can be predicted according to the previous work [31], which are related to the deviations of reflectivity |*r*_*p*_|^2^. Notably, such discrepancies also appear far away from the critical angle (namely $\theta_{i}\in \left[35{}^{\circ},45{}^{\circ}\right]$ ), as highlighted by the gray squares. In this region, the EMT is valid from the perspective of reflectivity in general. Such a breakdown of EMT stems from the ultra-sensitivity of the angular GH shifts on the reflectivity. The similar discrepancies also emerge for the spatial GH shifts and are up to 4 μm at the *θ*_*i*_ ≈ 40° far from the critical angle. It is ascribed to the deviation of Fresnel reflection coefficient phase *ϕ*_*p*_ which is closely related to the phase accumulation during the wave propagation. This process can occur in two ways: either the continuous way caused by the wave propagation in the medium, or the abrupt way induced by the wave reflection/transmission across the spatial boundary. The latter is sensitive to the stacking ordering, but the former is not. These phenomena indicate the strong dependence of the GH shift on the realistic stacking ordering of all-dielectric multilayers, even at the deep-subwavelength scale.

**Figure 3:**
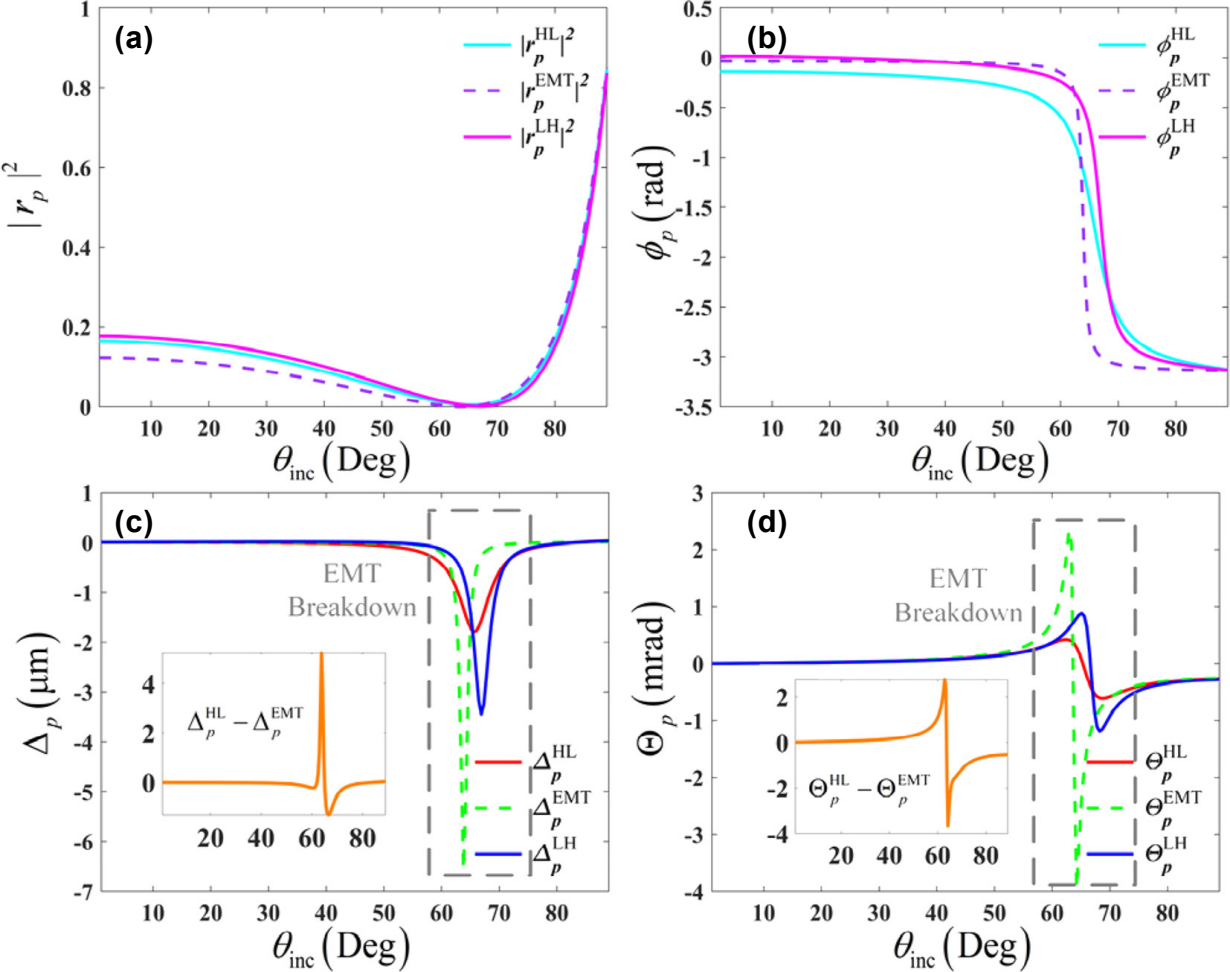
Breakdown of the EMT by the spatial (Δ) and angular (Θ) GH shifts on the all-dielectric periodic multilayered structures with different stacking orderings [*LH*] and [*HL*], in the case of *ɛ*_inc_ = 1. (a) Reflectivity |*r*_*p*_|^2^, (b) Fresnel reflection coefficient phase *ϕ*_*p*_, (c) spatial GH shifts (Δ), and (d) angular GH shifts (Θ).

In terms of the previous works [31], [32], [33], [34], the existence of a critical angle is a mandatory condition for the EMT breakdown in an all-dielectric system. Does it mean the GH shift calculated by the EMT is always exact in the all-dielectric structures without the critical angle? To tackle this issue, we replace the superstrate ZnSe prime in the setup shown in Figure 1(a) by the free space *ɛ*_inc_ = 1, and review the performances of reflectivity |*r*_*p*_|^2^, the reflection coefficient phase *ϕ*_*p*_, and GH shifts Δ and Θ in Figure 3. Interestingly, the discrepancies of the spatial and angular GH shifts persist as well in the angle range $\theta_{i}\in \left[60{}^{\circ},70{}^{\circ}\right]$ where no critical angle exist and the EMT is valid according to the reflectivity. Therefore, for the GH shifts strongly dependent on the phase and magnitude of the Fresnel reflection coefficient, the EMT can be disabled in any incident angles in principle. To summarize, Figures 2 and 3 illustrate that the EMT is not precise to homogenize the deep-sub-wavelength all-dielectric periodic multilayered structures when calculating the GH shifts, regardless of the existence of critical angle.

**Figure 4:**
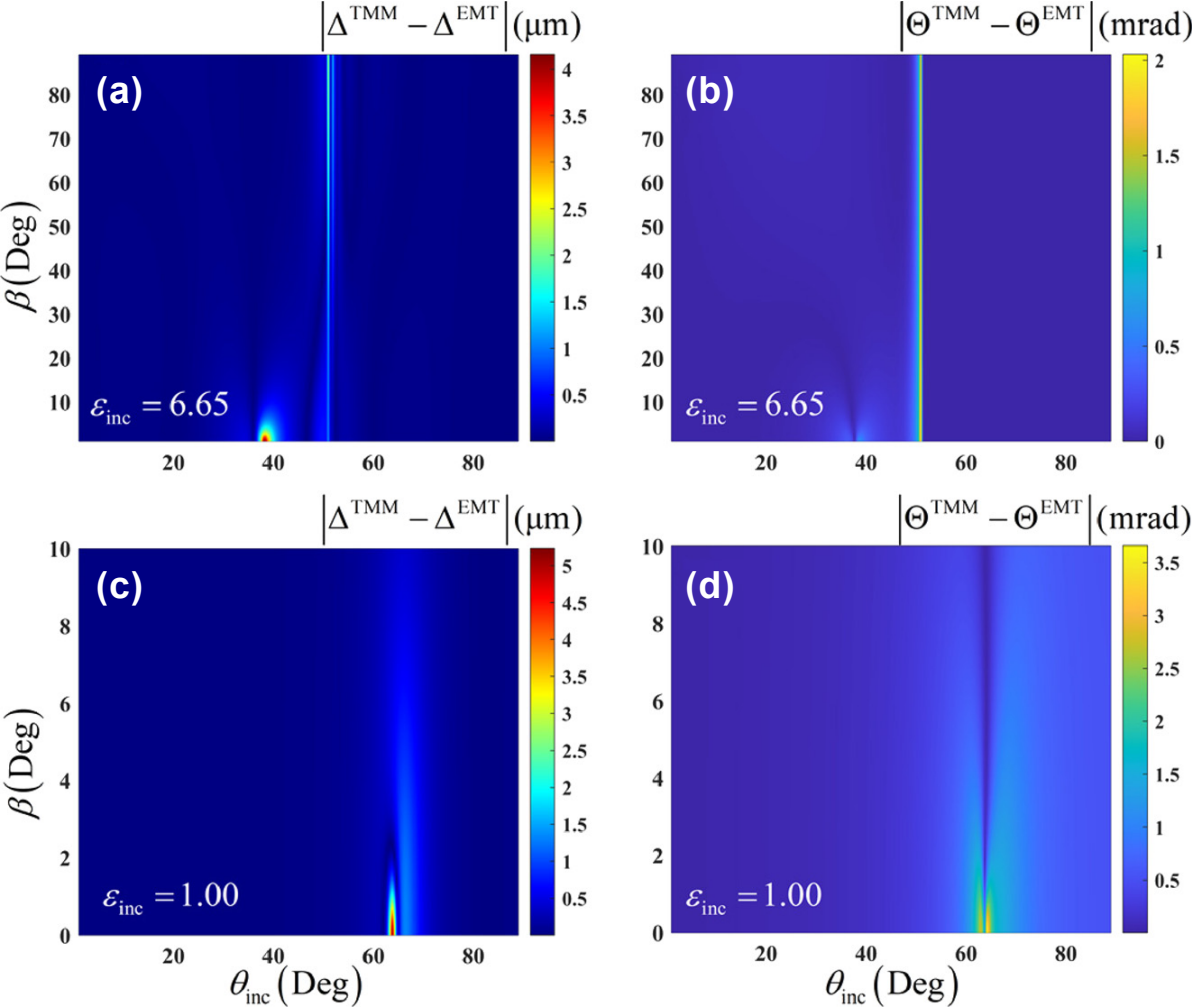
Influence of the polarization angle *β* on the performance of the EMT breakdown by the spatial (Δ) and angular (Θ) GH shifts, in the cases of (a), (b) *ɛ*_inc_ = 6.65, and (c), (d) *ɛ*_inc_ = 1.

Now, we proceed to examine the breakdown of the EMT by the spatial and angular GH shifts with different polarization angle *β* of incidence from Figure 4. As we can see, for the case of *ɛ*_inc_ = 6.65, the discrepancies in both spatial and angular GH shifts decrease with the growing polarization angle in regions far from the critical angle, whereas these discrepancies are robust against the variations of polarization angle near the critical angle. If the superstrate is free space, the errors of spatial and angular GH shifts under *p*-polarization have a pronounced peak near the incident angle *θ*_*i*_ ≈ 64°. But these errors rapidly diminish to near-zero values at larger polarization angles. These findings imply that the EMT achieves higher accuracy for larger polarization angles in the all-dielectric systems.

**Figure 5:**
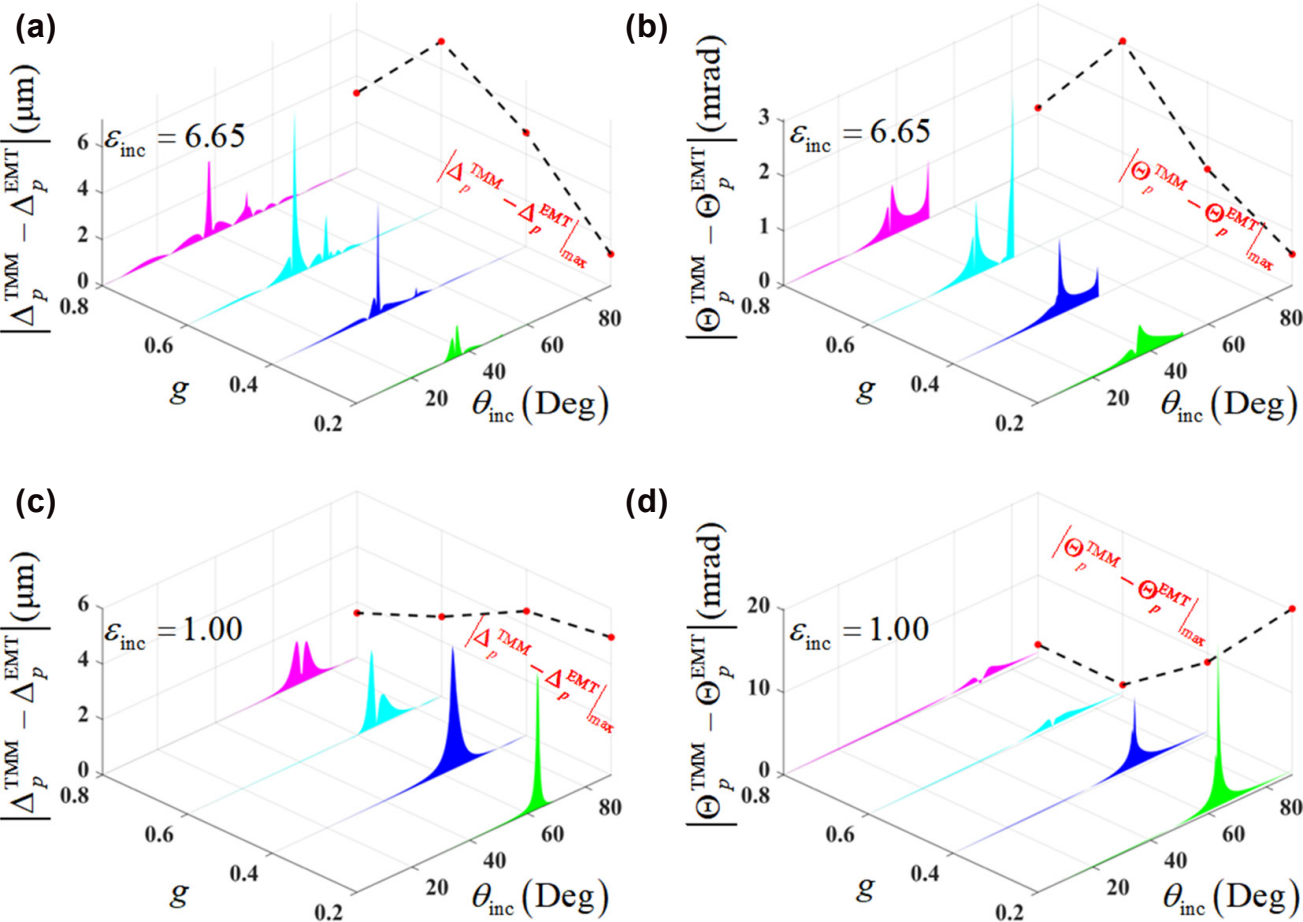
Influence of the filling fractions *g* = [0.2, 0.4, 0.6, 0.8] of structure on the performance of the EMT breakdown by the spatial (Δ) and angular (Θ) GH shifts, in the cases of (a), (b) *ɛ*_inc_ = 6.65, and (c), (d) *ɛ*_inc_ = 1. The filling fraction $g=d_{1}/\left(d_{1}+d_{2}\right)$ , and *d*_1_ + *d*_2_ = 20 nm is always satisfied.

Figure 5 depicts the breakdown of the EMT by the spatial and angular GH shifts with different filling fraction *g =* [0.2, 0.4, 0.6, 0.8] of the structure. The *g* is defined as $d_{1}/\left(d_{1}+d_{2}\right)$ and *d*_1_ + *d*_2_ = 20 nm is always satisfied. When a critical angle exists, the discrepancies of GH shifts will increase at first and then decrease as the filling fraction *g* rises, reaching the maximum value, when the thicknesses of the two components are nearly identical. It means that the EMT is more exact in the all-dielectric multilayers with large differences in the thicknesses of high- and low-index layers, when its superstrate has a high relative permittivity *ɛ*_inc_ > *ɛ*_⊥_. In systems without a critical angle, the discrepancies of GH shifts can be reduced via increasing the filling fraction *g*. Namely, increasing the proportion of the high-index layer in all-dielectric multilayers without the critical angle is helpful to weaken the degree of EMT breakdown.

**Figure 6:**
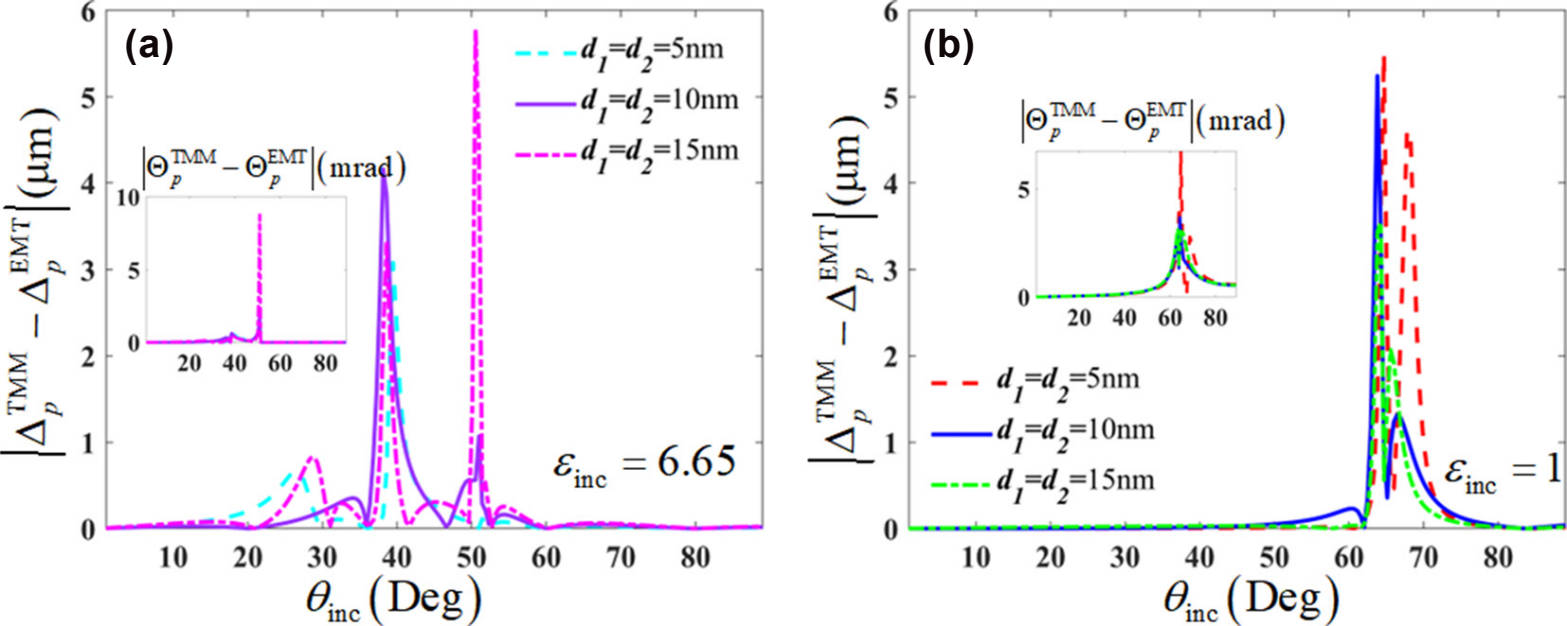
Influence of the unit-cell thickness on the performance of the EMT breakdown by the spatial (Δ) and angular (Θ) GH shifts, in the cases of (a) *ɛ*_inc_ = 6.65 and (b) *ɛ*_inc_ = 1.

Furthermore, we investigate the dependence of EMT breakdown induced by GH shifts on the unit-cell thickness, as shown in Figure 6. The results demonstrate that the EMT breakdown persists across a broad range of unit-cell thickness in the deep-subwavelength scale. For the case of *ɛ*_inc_ = 6.65, the discrepancies in both spatial and angular GH shifts become larger with the growing unit-cell thickness. In contrast, when *ɛ*_inc_ = 1, such deviations display a decreasing trend as the unit-cell thickness increases.

**Figure 7:**
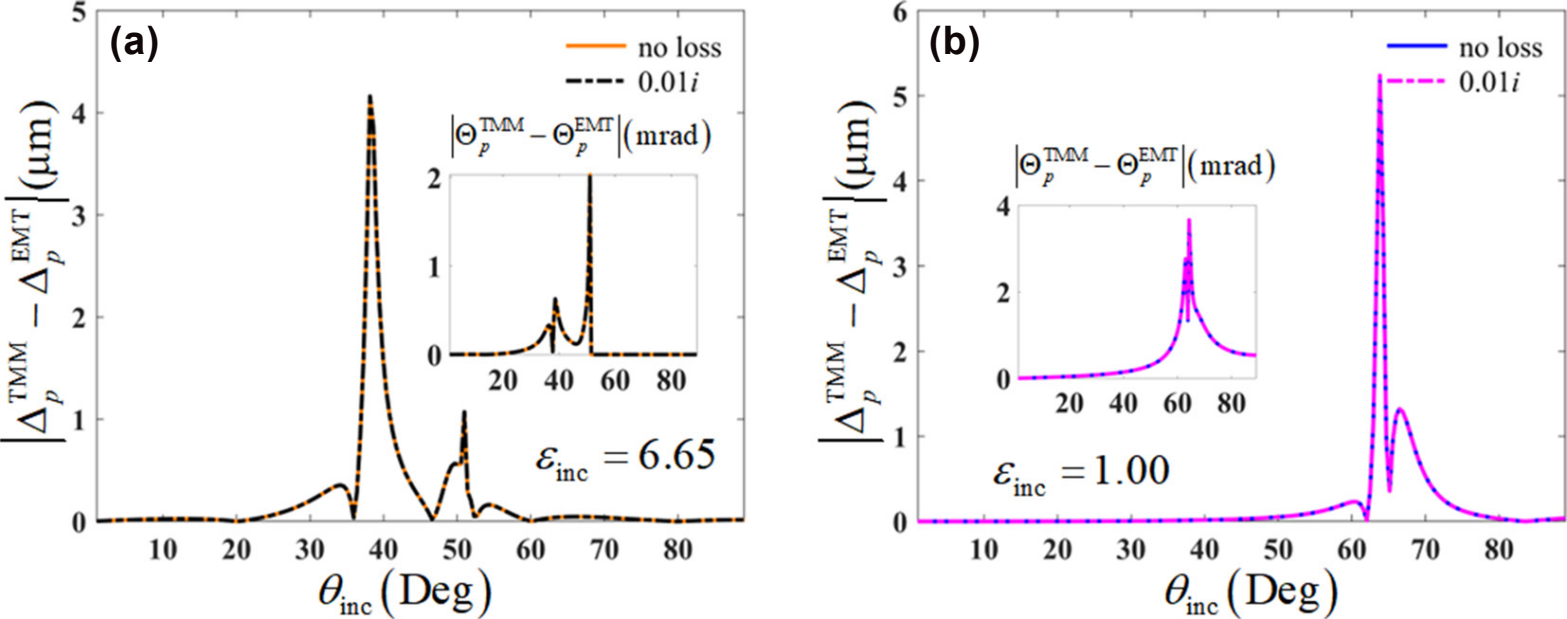
Influence of the multilayered components loss on the performance of the EMT breakdown by the spatial (Δ) and angular (Θ) GH shifts, in the cases of (a) *ɛ*_inc_ = 6.65 and (b) *ɛ*_inc_ = 1.

In the preparation of multilayered structures, the losses of materials are inevitable. That is to say, the relative permittivities of dielectric components are generally the complex numbers with tiny imaginary parts in the experiment. In Figure 7, we examine the effects of the losses of multilayered components on the EMT. It can be seen that, the spatial and angular GH shifts still invalidate the EMT for the loss cases, and the slight losses Imε1,2=0.01 on the dielectric components have few impacts on the discrepancies in both spatial and angular GH shifts. Hence, the conclusions in our work can be generalized to the cases for the lossy multilayered structures.

**Figure 8:**
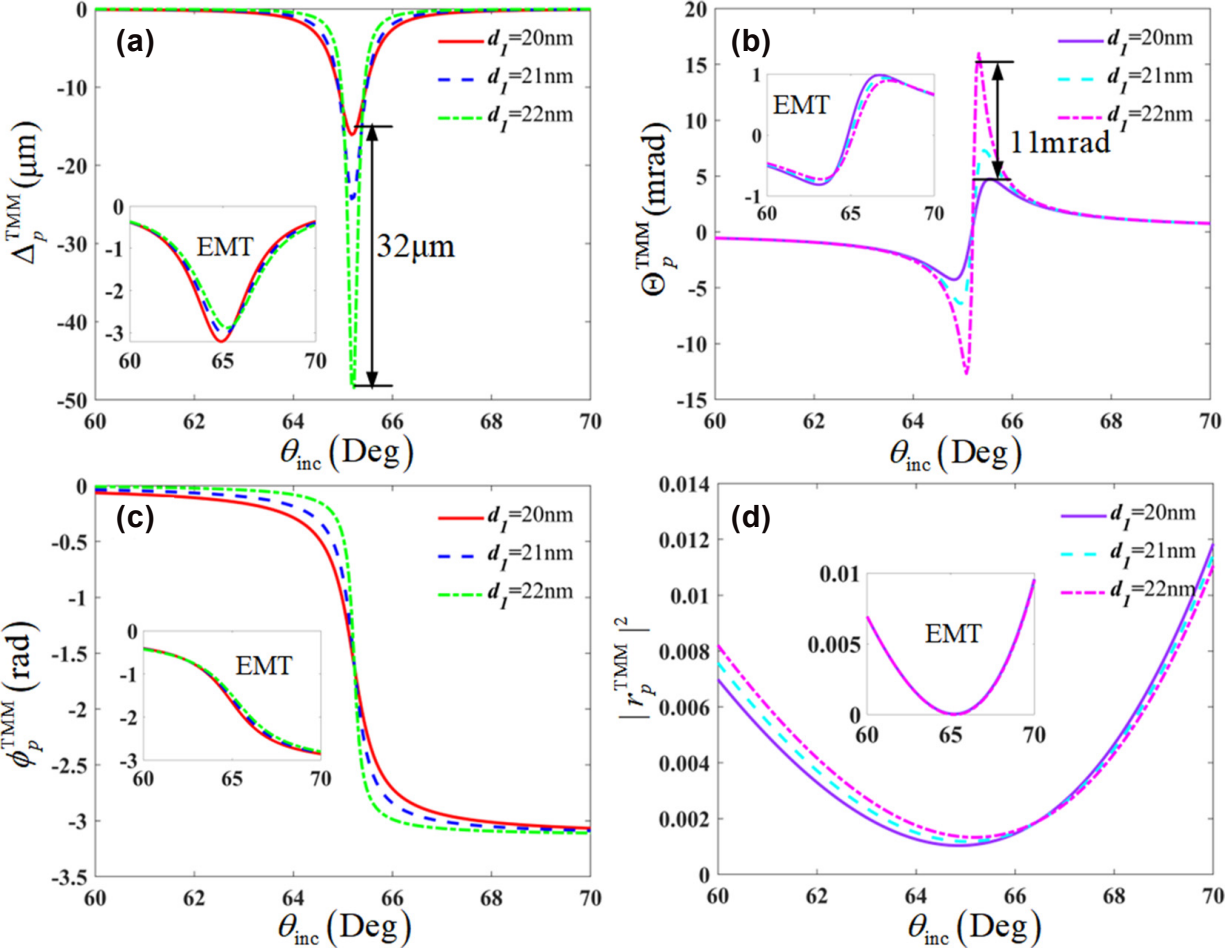
Breakdown of the EMT by the GH shifts with the stacking ordering [*HL*] in case of *ɛ*_inc_ = 1 and *N*_pair_ = 1, and the detection of dielectric structure defects at the extreme nanoscale. (a) spatial (Δ) GH shifts, (b) angular (Θ) GH shifts, (c) reflectivity |*r*_*p*_|^2^, and (d) Fresnel reflection coefficient phase *ϕ*_*p*_.

According to Ref. [31], the performance of EMT breakdown rely on the periodicity number *N*_pair_, and the severe breakdown phenomena happen only when *N*_pair_ ≫ 1. Here, we perform the breakdown of the EMT for case of *N*_pair_ = 1 by the spatial and angular Θ GH shifts with the stacking ordering [*HL*] and *ɛ*_inc_ = 1 in Figure 8. Although on the ultrathin slab with only 30∼33 nm thickness, the spatial and angular GH shifts still contradict to the conventional EMT. Importantly, only 1 nm variation in layer thickness can make very large distinctions in GH shifts. Whereas, such ultra-sensitivity on the thickness does not exhibit via the EMT. These differences can be explained by the reflectivity |*r*_*p*_|^2^ and reflection coefficient phase *ϕ*_*p*_. The 1 nm thickness change can hardly affect the |*r*_*p*_|^2^ and *ϕ*_*p*_ in the framework of EMT, but can be demonstrated via the TMM. It indicates that the homogenization in EMT may overlook the extreme small property variations of mediums. These phenomena indicate the GH shift is a powerful tool in the detection of dielectric structure defects at the extreme nanoscale, which has been overlooked before in the framework of EMT.

To experimentally validate the proposed concept, we employ the weak measurement [49] techniques in our setup. The experimental configuration begins with a Gaussian beam generated by the He–Ne laser passing through a Glan laser polarizer (GLP1) and a focusing lens (L1) before incident on the all-dielectric multilayered structures. The reflected beam then propagates through a quarter-wave plate (QWP), followed by a half-wave plate (HWP) and another Glan laser polarizer (GLP2), before being focused by a second lens (L2) onto a CCD detector for measurement. The breakdown of EMT can be experimentally demonstrated by comparing the measured GH shifts from all-dielectric multilayered structures with different stacking sequences. The weak measurement protocol enables significant amplification of the GH shift through careful selection of nearly orthogonal post-selection and preselection states. The multilayered structures, composed of alternating alumina (Al_2_O_3_) and titania (TiO_2_) layers with designed stacking orders, are fabricated using atomic layer deposition (ALD) to ensure precise thickness control and interfacial quality.

Finally, it is worthy to note that the EMT fails to predict the reflection phase in many cases, making it ineffective for GH shifts and other phase-sensitive physical phenomena. Therefore, the careful consideration of phase-related effects is essential when evaluating the applicability of EMT. It holds significant implications for the design of novel micro-nano optoelectronic devices.

## 4 Conclusions

In conclusion, we have reported the breakdown of EMT by the spatial and angular GH shifts on the periodic deep-subwavelength multilayered dielectric structures under the general conditions. Through comparing the GH shifts calculated by the EMT and TMM, it is found that the differences between them are very large in some cases commonly discussed. The underlying mechanism behind this phenomenon is ascribed to the high sensitivity of the GH shift on the Fresnel reflection coefficient phase and magnitude. Meanwhile, the numerical results have shown that the degree of the EMT breakdown can be controlled via adjusting the polarization angle of incidence and the layer and filling fraction of the structures. Finally, we have revealed the potential application of the GH shift on nano-meter scale thickness sensing, which may not be demonstrated within the EMT framework. This work will be helpful for improving the calculating accuracy of the electromagnetic responses and promoting the development of precise metrology devices.
